# The Perceived Value of Reducing Sedentary Behavior in the Truck Driving Population

**DOI:** 10.3389/fpubh.2019.00214

**Published:** 2019-08-07

**Authors:** Sarah L. Mullane, Douglas Connolly, Matthew P. Buman

**Affiliations:** ^1^School of Nutrition and Health Promotion, Arizona State University, Phoenix, AZ, United States; ^2^Stewart Transport, Phoenix, AZ, United States

**Keywords:** truck drivers, sedentary behavior, perceived value, Health Belief Model, engagement

## Abstract

**Purpose:** To conduct rapid qualitative analysis early in the intervention design process to establish the perceived value of reducing sedentary behavior in the truck driver population.

**Methods:** A rapid assessment process for qualitative data collection was used to examine managerial and employee perceptions quickly and iteratively to inform intervention design. Managerial insights were collected during semi-structured interviews and employee insights were collected via an online survey and focus group. Thematic analyses were guided by the constructs of the Health Belief Model to establish; (a) perceived susceptibility to the health problem; (b) perceived severity of the health problem; (c) perceived benefits of the potential solutions; (d) perceived barriers to adopting the recommended solution; (e) cues to action; and (f) self-efficacy.

**Results:** Three managers (2 females; 1 male) participated in semi-structured interviews. Seven truck drivers (1 female; 6 males) took part in a focus group. Sixteen survey responses (all male, mean age 49.8 ± 12.4 years, 86% white Caucasian) were collected in total (11 paper based; 6 online). The most important managerial motivators for engagement in an intervention included; improved sleep, alertness and quality of life. The most important employee motivators included; stress reduction (3.3 ± 1.3), improved quality of life (3.3 ± 1.3) and alertness (3.2 ± 1.4). Managerial and employee perspectives indicated that sedentary behavior may be of lower priority than diet and exercise, and may not resonate with the truck driving population as a health risk.

**Conclusion:** Application of the Health Belief Model indicated a disconnect between the researcher, managerial and employee perspective and the perceived value of a sedentary behavior reduction intervention. Within the truck driving population, researchers should endeavor to include safety as well as health outcomes, use multi-level strategies, design for outcomes of high perceived value and leverage health communication strategies to communicate benefits that resonate with the end-user.

## Introduction

Truck drivers are classified as one of the highest-risk occupational segments due to a complex interplay of health behavior barriers across the socioecological spectrum (1). In the commercial driving population, rates of obesity have been reported to be as high as 50%, while the prevalence of diabetes is 50% higher than the general population (2). Contributory factors include occupational influences on diet, exercise, sleep and more recently sedentary behaviors (1). Detrimental associations have been observed between prolonged sedentary time [any waking behavior in a seated or reclining posture with low energy expenditure (<1.5 metabolic equivalents [METS])] (3) and BMI (4), waist circumference and 2-hr plasma glucose (5). Bouts of standing (6–8), light-intensity physical activity (LPA) (7, 9–11) or non-exercise activity thermogenesis (NEAT) (12) may attenuate these effects. Despite a recent shift toward environmental changes (sit-stand/active workstations) in office-based work environment (13–15), further challenges exist in non-office environments where prolonged sitting is prevalent (e.g., occupational driver settings). Existing environmental, cultural and social solutions for sedentary office workers do not translate to this challenging setting and evidence on how to intervene is extremely limited (16).

Health promotion efforts within the truck driving population have primarily focused on exercise and/or diet based interventions (17). However, low engagement or high attrition rates are typically reported, which has led to concerns regarding sustainability and impact (18). Lack of engagement may be further amplified when targeting sedentary behavior reduction for two reasons. Firstly, the basic premise in ecological models of health behavior is that efforts to change individuals' behavior cannot be effective if environments make it difficult (19). As standing and moving is not conducive to occupational driving, it is likely the barriers are even higher than those associated with diet and exercise interventions. Secondly, health-related risk perceptions play an important role in motivating health behavior change (20). Although individual level factors such as family history may influence perceived susceptibility to disease, risk perceptions are often influenced by the frequency with which a threat is represented in media exposure (21). As an emerging health risk factor, which may not have substantive evidence and/or consistent media exposure, sedentary behavior may not be considered to be a “real” or high priority risk factor within the trucking population.

The Health Belief Model is a Cognitive Value-Expectancy Theory which emphasizes the perceived value of the outcome, and the subjective expectation that a behavior will result in the outcome (22). A person must feel personally susceptible to a disease with serious or severe consequences and must believe that the benefits of taking the preventive action outweigh the perceived barriers to (and/or costs of) preventive action, in order to adopt a recommended preventive health action. Personal values must be considered along with factual evidence of treatment efficacy in order to facilitate health promotion “*by the person*.” Determining how to intervene is contingent on the perceived susceptibility and perceived value of an intervention.

In an effort to address the disparate health risks of the truck driver population and to inform the development of interventions specifically targeting sedentary behavior, we seek to understand the perception of sedentary behavior in the truck driving population. The purpose of this research within the truck driving community was to examine the perceptions of sitting as a health risk factor. We have used the Health Belief Model as a guiding framework, including the barriers, motivators, the perceived value of and receptivity to potential health interventions, that may target sedentary behavior in the truck driving population.

## Methods

A rapid assessment process for qualitative data collection was used to develop a preliminary understanding of a situation from the insider's perspective quickly and iteratively to inform intervention design (23). Using a mixed-methods approach, formative research was conducted with a local Phoenix based truck company to retrospectively examine attitudes toward sedentary behaviors at work and the perceived opportunity for intervention (including barriers and motivators). An in-person, semi-structured interview (which allowed for expansion) was conducted by SM with managers of the trucking company to gather managerial insights. Employee insights were collected during an onsite visit to a driver training day which facilitated a focus group with active truck drivers. Due to the rapid assessment process and early stage of the partnership, detailed notes (rather than audio) were recorded (23). Finally, further employee insights were collected via a survey which could be completed online (via Qualtrics) or using a paper based version that was distributed in-person to truck drivers as they visited the headquarters. All responses were de-identified as soon as the response was recorded and a $10 gift card incentive delivered to each participant. In addition to demographic information, respondents were asked a series of 5-point Likert scale questions (not at all [1] to extremely [5]) to rate; (a) the likelihood of engaging in behaviors to reduce sitting; (b) the motivators for participating in an intervention to reduce sitting; and (c) the perceived value of supporting tools or mechanisms to increase engagement. This study was approved by the Arizona State University Institutional Review Board. All participants consented using an online or paper based consent form.

### Analyses

A thematic analysis of the key requirements and points raised during the semi-structured interviews and focus group was conducted. All closed survey question responses were imported and analyzed using SAS Enterprise Guide 7.1. All open ended questions were reviewed individually and word repetition used to identify the most prominent barriers and motivators (24). Findings were summarized using the constructs of the Health Belief Model (22) which included; (a) perceived susceptibility to the health problem; (b) perceived severity of the health problem; (c) perceived benefits of the potential solutions; (d) perceived barriers to adopting the recommended solution; (e) cues to action; and (f) self-efficacy.

## Results

**Figure 1:**
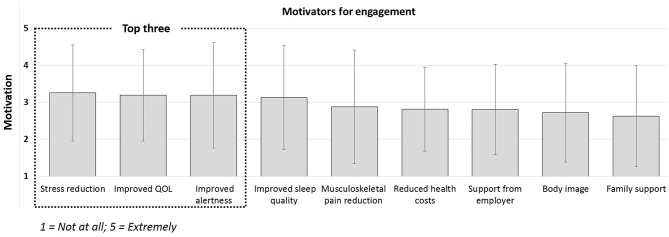
Employee motivators for engaging in preventative behaviors to reduce sedentary behavior (not at all [1] to extremely [5]).

**Figure 2:**
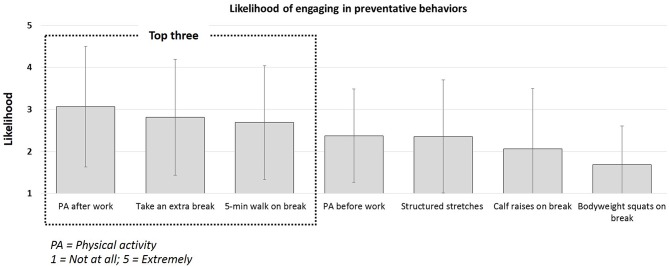
Employee likelihood of engaging in preventative behaviors to reduce sedentary behavior (not at all [1] to extremely likely [5]).

**Figure 3:**
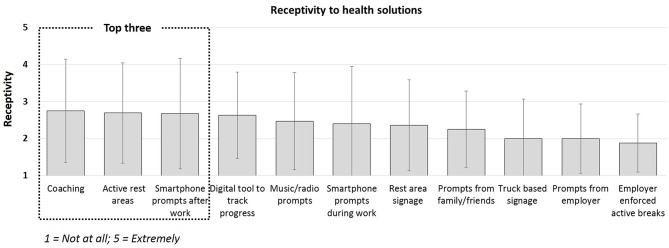
Employee receptivity to sedentary behavior reduction intervention components (not at all [1] to extremely [5]).

Three managers (2 females; 1 male) participated in semi-structured interviews. Seven truck drivers (1 female; 6 males) attended the driver training day and took part in a focus group. Sixteen survey responses were collected in total (11 paper based; 6 online), five of which were responses from those who also participated in the focus group. Of those that completed the survey, all were male, mean age 49.8 ± 12.4years and 86% white Caucasian. All survey and semi-structured interview findings are summarized in Table 1. Specific survey results pertaining to motivators for engagement, the likelihood of engaging in preventative behaviors and receptivity to suggested health solutions are presented in Figures 1–3, respectively.

**Table 1:** Managerial and employee perspectives categorized by the Health Belief Model constructs.

**Health Belief Model construct**	**Managerial perspective (I)**	**Employee perspective (F) (S)**
Perceived susceptibility	Voiced concern regarding the prevalence of diabetes and CVD risk in the trucking population	Highly aware of a detrimental relationship between prolonged sitting and musculoskeletal pain
	Awareness that sitting may contribute to health risk but see lack of overall exercise and poor nutrition as the main culprits	Voiced concern regarding unhealthy diet choices due to trucking rest areas and susceptibility to diabetes
	Do not necessarily see strong link between poor glucose control and sitting, i.e., awareness that sitting may contribute to health risk but see lack of overall exercise and poor nutrition as the main contributors to poor health	Acknowledged lack of time to exercise routinely, particularly when on the road and that this may contribute to diabetes, weight gain and overall poorer cardiometabolic health.
	Acknowledgment of truck driver health being an area for concern.	
Perceived severity	Highest priority is the cyclical relationship between poor health, poor sleep and resultant alertness on the road.	Concern regardng the prevalence of obesity and diabetes in truck driver population but attributed this to diet and exercise (not sitting) (F)
	Voiced concern regarding the impact of diabetes in the trucking population	
	Acknowledgment of the detrimental impact of an aging truck driver population and poor health on the ability to recruit new truck drivers into the profession	
Perceived benefits of solutions	Improved driver safety	Reduced stress (S)
	Improved professional reputation and resultant employee recruitment	Improved QOL (S)
	Improved driver health	Improved sleep and alertness (S)
	Improved driver QOL	Reduced MSK pain (F)
Perceived barriers to solutions	Uncertainty regarding the effectiveness of smartphone solutions which may serve as a distraction while driving	Lack of time during break
	Driver regulations and policies i.e., 30 min break, 10 h clock.	Lack of safe parking which may cause them to drive for longer
	Truck driving culture and aging population	Being “on the clock”
	Time	*The 14h clock. Work sched/type does not allow such thoughts. We are paid by the mile. We have X amount of time to get all the miles we can. Miles trump everything when you are paid by the mile.'*
	Isolated nature of driving. Limite contact with employees	
	Disconnect between researcher and real world	
Cues to action	Solution that also targets sleep and alertness outcomes	Coaching (S)
	Rest area redesign	Active rest areas (S)
	Signage	Smartphone prompts after work (S)
	Coaching	Interactive tool to track overall health (S)
		Tool that incorporates parking and rest area infromation (F)
Self efficacy	During a shift = **Low** due to the need to drive while seated, and work long hours. May require policy changes.	During a shift = **Low** due to the need to drive while seated, and work long hours. May require policy changes (F)
	Before or after a shift = **High** due to increeased opportunity outside of truck envronment and time constraints.	Before or after a shift = **Medium** due to increeased opportunity but still conflicts with other daily life stresses and responsibilities (F)
		If given the choice at work, truck drivers reported that they would:
		Sit 55% of the time
		Stand 15% of the time
		Move 30% of the time (S)

*Data collection type denoted as Interview (I), Focus group (F) or Survey (S)*.

### Manager Perspective

The results presented in Table 1 (employer perspective) indicated that managers acknowledged high susceptibility to diabetes and overall increased cardiovascular disease (CVD) risk in the truck driver population compared to the general population. However, they did not necessarily attribute this risk to prolonged sitting but instead a combination of poor diet and lack of exercise due to being “on the road.” Perceived severity was considered high due to their concern regarding the cyclical relationship between poor health, poor sleep and alertness and potential impact on driver safety. Perceived benefits were consistently related to driver safety by potentially improving sleep, alertness, well-being and quality of life. A notable managerial perceived benefit included the ability to change the public perception of the profession as being detrimental to health, which may facilitate future recruitment efforts. Perceived barriers included the potential for driver distraction that may arise via digital solutions and existing policies which dictated work breaks and time on the road. Additional concerns were raised regarding the isolated nature of driving and being able to distribute change across the organization with limited group based contact. Finally, concerns were also raised regarding the disconnect between research and the real world and the extent to which the driving culture may override researcher efforts.

Managerial cues to action included an intervention designed to monitor sleep and alertness, in addition to glucose control. Rest area redesign, signage and coaching were identified as being desirable components of the health solution. Digital solutions were only deemed appealing if they targeted rest areas, breaks and out of work hours (i.e., not while driving). Overall perceived self-efficacy of being able to reduce prolonged sitting and increase levels of activity during the work day was low given the nature of the job. However, targeting outside work hours was considered much more feasible.

### Employee Perspective

The results presented in Table 1 (employee perspective) indicate that employees voiced stronger susceptibility concerns regarding the detrimental effects of sitting but as a contributing factor to musculoskeletal pain rather than diabetes. Similar to managers, they attributed diabetes and CVD risk to poor diet choices and lack of exercise “on the road.” Although truck drivers recognized the prevalence of diabetes and CVD risk in their profession, perceived severity was lower than the manager perspective. More emphasis was placed on the severity and detrimental effects of stress on the truck driver population. Perceived benefits derived from the survey responses (see Figure 1) indicated that stress reduction (3.3 ± 1.3) was the most important motivator and benefit of engagement. Additionally, improved quality of life (3.3 ± 1.3), alertness (3.2 ± 1.4), sleep (3.1 ± 1.4) and pain reduction (2.8 ± 1.5) were considered important motivators. The least important motivator was identified as family support (2.6 ± 1.4). Perceived barriers were similar to those voiced by the managers with an additional concern regarding the availability of safe parking areas which, in some cases, causes drivers to drive for longer than planned. Lack of time to engage in any health solution was consistently identified in both group discussion and survey responses as a significant barrier.

Cues to action identified from survey responses (see Figure 2) indicated that participants were most receptive to engaging in physical activity after work (3.1 ± 1.4) but least likely to engage in bodyweight squats during a break to reduce sedentary behavior (1.7 ± 0.8). The survey responses presented in Figure 3, indicated that participants were most receptive to the idea of health coaching (2.8 ± 1.4), active rest areas (2.7 ± 1.4) and smartphone prompts after work (2.7 ± 1.5). They were least receptive to employer enforced active breaks (1.9 ± 0.8). Finally, employees reported that if they were given the opportunity, they would prefer to sit (54.6 ± 38.9%), stand (15.4 ± 23.6%) and move (30.0 ± 31.4%) of the time during their working day. However, the perceived ability to do so was low during the work day, and slightly higher outside of work hours.

## Discussion

Efforts to promote health are likely to be ineffective if they ignore what a person values, which influences the appraisal of the risk-benefit ratio for different treatments or lifestyle practices (25). Application of the Health Belief Model indicated that there was a disconnect between the perceived value of an intervention designed to reduce sedentary behavior between the researcher, managerial and employee perspective. Based on these findings, pertinent insights that may increase perceived value and future engagement in truck driving health interventions are outlined below.

### Leveraging Safety as Well as Health

Our results support existing evidence advocating for health interventions in the trucking population that integrate occupational safety with health promotion (26). Initially, managerial hesitancy toward a digital intervention was apparent due to possible driver distraction. However, this hesitancy was reduced when considering the possibility of targeting sleep and alertness outcomes, for which the perceived value was high. Accumulating evidence suggests that obesity (27) and insulin resistance (28) may play a significant role in the pathogenesis of excessive daytime sleepiness (EDS) which is associated with “drowsy driving” and may result in 1,500 road deaths and 40,000 injuries annually (27, 29). Interestingly, research has also indicated that intermittent bouts of LPA have been linked to increased alertness (30, 31). Leveraging this evidence and incorporating it into a multi-component intervention may increase the perceived value within the trucking driving community. To increase perceived value from all parties, future interventions should consider additional measures of sleep and alertness (in addition to activity levels and glycemic control).

### Social Ecological Determinants of Health

Both employers and employees voiced concern regarding factors outside their control- hours of service laws and parking availability at either rest areas or truck stops which dampened any efforts to engage in health interventions. These results support the notion that determinants of health extend beyond individual lifestyle and health services factors, with social, economic, organizational, environmental, and cultural factors all determinants in health (17). Ecological domains, such as governmental, corporate, organizational, community, and built-environment factors, can support or inhibit opportunities and resources available for physical and recreational activities (32, 33). Alternatively, both managers and employees expressed increased perceived value in efforts to promote active rest areas. This is in support of previous research which identified highway rest areas as the highest scoring active living setting for the truck driver population (35.3%) compared to warehouses which were identified as the lowest scoring (11.6%). We recommend that researchers engage multiple stakeholders representing key user personas early in the design process. This may help identify motivators and barriers at the individual, social, cultural and organizational level, and facilitate translation into real world settings.

### The Importance of the End-User

Emerging theories suggest that lack of user involvement early in the intervention design process has been identified as one of the major contemporary difficulties encountered during intervention implementation (34). While it is important to acknowledge the high perceived value of safety from the managerial perspective as a motivator to facilitate health intervention for employees, it is important to not lose sight of those who will ultimately participate- i.e., truck drivers. Stress reduction and improved quality of life were perceived as the greater motivators and may therefore be a key component for engagement. We recommend that researchers be creative in the design of an intervention to incorporate higher priority factors as perceived by the end-user, rather than focusing on the researcher and/or stakeholder primary aim alone, e.g., improved glycemic control or alertness.

### Applying Health Communication Theory

In addition to designing for the end-user, researchers may need to incorporate Health Communication theory to better communicate benefits that resonate with the target population (35). The power of effective communication and “framing,” or conversely, the impact of miscommunication, is reflected in our results. Conflicting priorities are evident between the likelihood of engagement in behaviors (Figure 2) and receptivity to potential health solutions (Figure 3). Although “taking an extra break” was identified by employees as the second most likely behavior they would engage in to reduce sedentary behavior, “employer enforced active breaks” was the least preferred health solution. The terminology “enforced” eludes to having less control over the working day and although it would indeed reduce sedentary behavior, the negative perception of less control from the employee perspective may impact employee morale, cause more stress and reduce quality of life. We posit that redefining this option as “support from employer to take an active break,” may have elicited a more favorable result. Researchers should be encouraged to consider the unintended consequences of health solutions and health communication, which although may align with researchers and stakeholder goals, may detrimentally impact the end-user perception (36).

### Measures and Method of Data Collection

Truck driving is reported as a primarily isolated profession that does not require regular group based contact with work colleagues (17). Therefore, the employer has intermittent contact with each employee. Only ~32% of the surveys were completed online, the rest were completed on paper. Email communication is not required for the job and thus, the employer does not have an email address for all employees. This reduces the opportunity for interaction with employees and may impact both recruitment and assessment strategies for future trials. Only by gaining trust and through support from the employer were we able to collect a small number of survey responses. The difficulty experienced during recruitment, is reflective of the challenges faced in previous studies reporting low engagement or high attrition (1, 17), and further highlights the need to incorporate our recommendations to; include safety as well as health outcomes, use multi-level strategies, design for outcomes of greater perceived value and communicate the benefits that resonate with the end-user.

## Limitations

We acknowledge that these insights are not generalizable given the small sample size. Similarly, formative research was not audio recorded and there was little variation across the receptivity and motivators for engagement results. However, using rapid assessment processes, we obtained early user feedback quickly and cost-effectively, that may significantly impact intervention design. Too often, researchers do not provide enough insight regarding user research and resultant design decision-making process that may help to inform future research. This is supported by Yeager et al. (37), who recently proposed a framework for “Design Thinking for Psychosocial Interventions” defining different “lenses” to incorporate users i.e., participants, communities, stakeholders etc., early in the intervention design process to avoid easily discoverable flaws that impede real world application (37). Such novel insights are also shared by Community-Based Participatory Research (CBPR)-which aims to unite health professionals, academics, and communities in giving underserved communities a genuine voice in research to increase the likelihood of an intervention's success (38). An important facet of CBPR is the identification of “gate keepers” within the community- without their support and collaboration, an intervention is likely to fail regardless of community demand (38). Our formative research has nurtured relationships and fostered negotiation to provide a more sustainable partnership that can support real world application.

## Conclusions

Truck drivers are exposed to prolonged sitting and rest areas are not perceived as “healthy” environments, and the associations between, obesity and insulin resistance could be fatal. However, the disconnect between what a researcher may perceive as a valuable intervention may not be reflected in the target population. Establishing perceived value is critical to health solution dissemination and true health impact and may require some negotiation in order to gain “buy-in” (not necessarily consensus) from all parties involved. Identifying motivators to participation may impact the intervention measures and communication of the study and should be considered when designing within the community. Such findings can be elicited quickly, cost-effectively and early in the design process using rapid assessment processes to collect and analyze qualitative data. Our findings indicated that while prolonged sitting may be considered a major topic within research, it is yet to resonate with the truck driving population as a “health risk.” Although a researcher may endeavor to target a specific behavior, if the value of the perceived intervention is not high enough, it is unlikely that the participant will continue to engage in the preventative behavior. The ability to design sustainable interventions is crucial to public health impact and must be aligned with perceived value of the intervention. There is continued need for user feedback from truck drivers and associated stakeholders to understand the perception of sedentary behavior and potential receptivity to reducing it, to improve the level of engagement and resultant effectiveness of future trials.

## Ethics Statement

This study was approved by the Arizona State University Institutional Review Board and all participants consented using an online or paper based consent form.

## Author Contributions

SM was responsible for the concept and design of the study. SM and DC were primarily responsible for data collection. SM performed data analysis and interpreted the data. SM wrote the initial draft of the manuscript. SM, MB, and DC reviewed and edited the manuscript, and approved the final version prior to submission. All authors reviewed and approved the final manuscript as submitted.

### Conflict of Interest Statement

The authors declare that the research was conducted in the absence of any commercial or financial relationships that could be construed as a potential conflict of interest.
